# Comment on “Effect of Deep Slow Breathing on Pain-Related Variables in Osteoarthritis”

**DOI:** 10.1155/2021/6384191

**Published:** 2021-02-16

**Authors:** Dr. Meenu Verma, Dr. Shilpasree Saha

**Affiliations:** Maharishi Markandeshwar Institute of Physiotherapy & Rehabilitation, Maharishi Markandeshwar Deemed to be University, Mullana, Ambala, Haryana 133207, India

We would like to congratulate Larsen et al. for their valuable work published in *Pain Research and Management* titled “Effect of Deep Slow Breathing on Pain-Related Variables in Osteoarthritis” [1]. The current study is informative and may help in clinical practice to improve pain and physical function in patients with osteoarthritis. However, there are some methodological issues in this paper which need to be highlighted.

Firstly, the authors did not mention how they had estimated the sample size. Sample size estimation is necessary for approval or rejection of results of clinical trial irrespective of how clinically effective or ineffective the intervention may be. In instances where sample size selected is less than what is required, this may cause statistically nonsignificant results, even though clinical significance exists. If the selected sample size is much more than what is required, then even a small difference between two interventions will produce a statistically significant result, even if the difference is not clinically meaningful [2].

Next, in the section of “Data Collection and Analysis,” the authors did not mention the use of a normality test in their article. They directly mentioned that the variables were analysed using three separate mixed ANOVAs. Statistical analysis of data is always dependent on a normality test which can be checked by the Kolmogorov–Smirnov (K–S) test and Shapiro–Wilk test in SPSS. Depending on the normality distribution, parametric and nonparametric tests are used to analyse changes in values of variables [3]. Appropriate statistical tests are important in clinical research to ensure results are interpreted correctly.

We are thankful to the authors for their valuable research, and we would like to suggest the authors consider the issues discussed above before applying their results to clinical practice.
